# User testing a patient information resource about potential complications of vaginally inserted synthetic mesh

**DOI:** 10.1186/s12905-020-01166-4

**Published:** 2021-01-25

**Authors:** Nikolina Angelova, Louise Taylor, Lorna McKee, Naomi Fearns, Tracey Mitchell

**Affiliations:** 1 Health Services Researcher, Healthcare Improvement Scotland, Gyle Square, 1 South Gyle Crescent, Edinburgh, EH12 9EB Scotland, UK; 2 Information Analyst, Healthcare Improvement Scotland, Gyle Square, 1 South Gyle Crescent, Edinburgh, EH12 9EB Scotland, UK; 3 Emeritus Professor of Management and Health Services Research, University of Aberdeen, 3rd Floor, Health Sciences Building, Foresterhill, Aberdeen, AB25 2ZD Scotland, UK; 4 Healthcare Improvement Scotland, Delta House, 50 West Nile Street, Glasgow, G1 2NP Scotland, UK

**Keywords:** Vaginal mesh implants, Mesh complications, Stress urinary incontinence, Pelvic organ prolapse, Patient information leaflet

## Abstract

**Background:**

Vaginal mesh implants are medical devices used in a number of operations to treat stress urinary incontinence and pelvic organ prolapse. Although many of these operations have delivered good outcomes, some women have experienced serious complications that have profoundly affected their quality of life. To ensure that evolving patient information is up-to-date, accurate and appropriate, the Transvaginal Mesh Oversight Group ‘user-tested’ a newly developed Scottish patient resource, the first to focus exclusively on the issue of complications. The aim of this research was to gather feedback on usability, content, language and presentation to inform the development of the resource from a user perspective.

**Methods:**

The experience of using the patient resource was captured through semi-structured interviews that followed a ‘think-aloud’ protocol. The interviewer observed each participant as they went through the resource, asking questions and making field notes. Participants’ comments were then categorised using a validated model of user experience and subsequently analysed thematically.

**Results:**

Thirteen people participated in the user testing interviews, including women with lived experience of mesh implants (n = 7), a convenience sample of staff working for Healthcare Improvement Scotland (n = 5) and a patient’s carer (n = 1). The majority of participants considered the resource as clear and helpful. Respondents reported that some presentational aspects promoted usability and understandability, including the use of a font that is easy to read, bullet lists, coloured headings and simple language. Barriers included the reliance on some technical language and an explicit anatomical diagram. Participants endorsed the valuable role of health professionals as co-mediators of patient information.

**Conclusions:**

The findings illustrate the value of undertaking in-depth user-testing for patient information resources before their dissemination. The study highlighted how the direct guidance or navigation of a patient information resource by a health professional could increase its salience and accuracy of interpretation by patients, their families and carers. These insights may also be useful to other developers in improving patient information.

## Background

Stress urinary incontinence (SUI) and pelvic organ prolapse (POP) are common conditions that can have a profound impact on the physical, psychological and social wellbeing of women and their families [1, 2]. SUI is characterised by leaking of small amounts of urine with activities that increase abdominal pressure such as coughing, sneezing, laughing or exercising. POP is a condition when a prolapse arises in one or more of the organs in the pelvis. Based on the successful use of synthetic mesh in other surgical fields, surgical procedures using a vaginal mesh implant have been developed to treat both SUI and POP.

Between April 2009 and March 2019, 8384 mesh procedures for SUI and 1519 mesh procedures for POP were undertaken in Scotland [3]. For many women, these operations have delivered good outcomes. However, some women have experienced serious complications that have left them unable to participate in family, work and social life [4, 5]. Some adverse events associated with the use of synthetic meshes for prolapse and incontinence may include vaginal exposure, pain, infection, bleeding, erosion into adjacent organs, mesh shrinkage and/or organ perforation [5]. It is not known exactly how many patients have experienced these kind of complications, which has contributed to divided opinion on the safety of vaginal mesh implants.

In 2017, New Zealand became the first country to ban the use of all surgical mesh products for transvaginal POP repair and a single incision mini-sling for the treatment of SUI, when the country’s Ministry of Health asked transvaginal mesh suppliers to stop selling their products [6]. Other products, such as mesh tapes used in mid-urethral sling operations for SUI, required changes to their Instructions for Use to amend the indications and/or add warnings before they could continue to be supplied in New Zealand.

To get a better understanding of the problem, the Scottish Government launched an independent review of transvaginal mesh products in 2014 and recommended that the use of these products for SUI and POP be suspended until more evidence was gathered. The independent review recommended against the routine use of transvaginal mesh in the treatment of POP [5], although some health boards continued to perform certain procedures for SUI until 2018. In February 2018 the Secretary of State for Health and Social Care announced an Independent Medicines and Medical Devices Safety Review (led by Baroness Julia Cumberlege), to consider the use of transvaginal mesh, among other health technologies [7]. As part of this review Baroness Cumberlege met with women (and their families) who had been adversely affected by procedures that use transvaginal mesh and subsequently recommended a halt to the transvaginal mesh procedures for SUI [8]. In response, the Scottish government instructed health boards to halt all mesh procedures in September 2018, pending the development of a ‘Restricted Use Protocol’ [9], which was still in development at the time of writing this paper. According to the latest data review provided by Information Services Division (ISD) Scotland, no mesh procedures for SUI and POP were carried out between October 2018 and March 2019 since the halt was announced [3]. Other surgical procedures offered in Scotland if a non-surgical management for SUI has failed include colposustension and autologous rectus fascial sling.

Following the independent review, the Scottish Government tasked Healthcare Improvement Scotland (HIS) to establish an independent multidisciplinary Transvaginal mesh implant oversight (TVMO) group to monitor the use of transvaginal mesh in Scotland. One part of their remit was to review the range and quality of information relating to transvaginal mesh available to NHSScotland patients to investigate whether it is up-to-date, accurate and appropriate. To do this, the TVMO group established a multidisciplinary patient resource sub-group to identify and quality assure the available patient information resources. The group included clinicians, health services researchers and patient representatives with lived experiences, one of whom acted as its Co-Chair.

The Knowledge Management team in HIS systematically searched for existing patient information resources and identified a total of 61 patient-focussed resources on SUI or POP produced by NHS organisations and other UK and international English speaking professional associations/societies. The patient resource sub-group then quality assured these existing resources using a DISCERN tool [10], which is a validated questionnaire that can be used to assess the quality of written patient information. The group gave feedback to the organisations involved in the production of the resources that scored poorly. Following this quality assurance, user testing was undertaken to further quality assure two patient resources: one was about physiotherapy interventions for SUI and POP and the other was about potential complications after surgical mesh procedures for the treatment of SUI and POP.

This paper focuses exclusively on the user testing of the complications resource [11], titled *Vaginally Inserted Synthetic Mesh: Potential Complications* (see Additional file 1).

There are two main reasons for this focus. Firstly, the DISCERN review identified several well-developed physiotherapy leaflets but highlighted a dearth of high quality information resources for women who have previously had a synthetic mesh inserted into the vagina and are concerned about potential mesh complications. To address the emerging information gap, a Subspecialist Urogynaecologist from NHS Lothian (member of the TVMO board and patient resource sub-group) led the development of the complications resource, which explains in detail the potential complications of vaginally inserted mesh for POP or SUI, what women should do if they are experiencing these complications, information about mesh removal and the risks related to it. Secondly, given that the complications resource was at an early pilot stage and thus not yet in circulation, there was a unique opportunity to modify, improve and add value to the resource guided by direct end user input.

The aim of this paper is to synthesise feedback from the user testing exercise of the mesh complications resource, including insights on the usability, content, language, presentation and means of sharing this new resource. It identifies areas for improvement and discusses how such a resource might be made useful both for women with lived experiences, their family and carers and the wider public. The paper also discusses some generic points about usability of patient information resources and the co-navigation role of health care professionals (HCPs) in use of such materials. It suggests that where the topic is sensitive and/or complex, close engagement with health professionals in using the resource can be valuable.

## Methods

### User testing

User testing was chosen as robust method for evaluating patient information and informing improvements [12–14]. For example, findings from user testing of a patient version of a Scottish Intercollegiate Guideline Network guideline for glaucoma [15] helped inform improvements to the resource from a user perspective [16]. This informed the current choice of method and design.

The experience of using the resource was captured through semi-structured interviews that followed the ‘think-aloud’ protocol developed by Rosenbaum [17]. In this approach, the interviewer observes the participant as they read through a resource and encourages them to articulate their thoughts about how they understand and experience the information. This allows the tester to gain an understanding of the user’s experience, observe any issues they encounter and obtain any suggestions for improvements.

The user interviews were conducted by a HIS Project Officer (TM) who used an amended interview guide originally developed by Fearns et al. (2016) (see the adapted guide Appendix 1). The interviewer presented the complications resource to each participant as a hard copy on A4 paper. Each interview lasted approximately one hour. All interviews were audio recorded and transcribed by TM, who also took field notes in order to document observations and contextual information [18].

### Participants and setting

User testing interviews were conducted between January and March 2019 at widespread locations across Scotland, including Glasgow, Aberdeen, Dumfries and Shetland. It was estimated that a sample of 12–15 participants would be sufficient to achieve saturation, based on the user testing conducted by Fearns et al. [16].

Initially a purposive sample of patients who had undergone operations using vaginal mesh implants for SUI and POP were recruited through the HIS Patient and Public Involvement unit. In light of the highly sensitive nature of the subject matter, social media was not considered an appropriate tool for recruitment, therefore the Public and Patient Involvement unit approached third sector organisations (including Health and Social Care Alliance Scotland, Bladder and Bowel Community and Bladder Health UK) to invite patients to participate. Participants were also recruited through networking opportunities at a menopause conference. Seven women with lived experience of transvaginal mesh and one carer were successfully recruited in this way but despite extensive efforts the response rate remained low.

Recognising the low response rate and the high profile of transvaginal mesh at the time of the study, the research team adopted an alternative recruitment approach to include a broader range of women with no direct mesh experience. An invitation to participate was extended to women staff working in different departments of HIS as personal assistant, administrative officer, project officer, programme manager, quality improvement advisor and a public partner. The aim of using a convenience sample was to recruit participants with a broad and independent range of perspectives and different degrees of knowledge and experience. Previous studies of user-testing of patient information leaflets have also included a convenience sample of health professionals [19].

### Analysis

A Research Analyst (LT) analysed participants’ feedback using a framework based on Morville’s honeycomb model [20]. Morville’s original model, which describes seven facets of the user experience, was adapted by Rosenbaum [17] for the evaluation of evidence-based resources in the healthcare setting. The adapted framework describes eight facets of user experience: accessibility, findability, usefulness, usability, understandability, credibility, desirability and affiliation.

After familiarisation with the interview transcripts, LT grouped participants’ comments into the categories of the framework. To ensure participants’ comments were interpreted correctly, LT and TM discussed any areas of ambiguity and TM used her field notes to provide clarity. LT then conducted a thematic analysis to explore themes within seven of the eight categories: *findability* was not included in the analysis because of the way in which participants were presented with the resource. A second researcher (NA) coded a cross section of the interview transcripts to check for consistency and promote rigour. Inconsistencies were discussed until agreement was reached.

## Results

Thirteen people agreed to participate in the user testing interviews, including patients (n = 7), HIS staff (n = 5) and a patients’ carer (n = 1). All participants were women who were native English speakers residing in Scotland. Participants had varying levels of education and were aged between 26 and 81. The distribution of participants by socio-demographic characteristics is presented in Table 1. To preserve anonymity, the names published in this paper are pseudonyms.

**Table 1:** Participant demographics

Name	Age range	Education level	Patient, staff or career
Belinda	40–49	University Degree	Patient
Claudia	50–59	High School	Patient
Daisy	80–89	University Degree	Patient
Gillian	50–59	High School	Patient
Jessica	60–69	University Degree	Patient
Louise	40–49	University Degree	Patient
Orla	60–69	High School	Patient
Anna	50–59	Diploma	Staff
Fiona	40–49	University Degree	Staff
Hillary	50–59	Postgraduate Degree	Staff
Ingrid	50–59	PhD	Staff
Kate	40–49	University Degree	Staff
Emma	20–29	University Degree	Carer

Overall, the majority of participants considered the resource to be clear and helpful, although participants’ opinions and preferences varied considerably. Differences in opinion did not appear to be related to the participants’ age, level of education or whether they were a patient or a staff member. A summary of the findings is presented in Table 2.

**Table 2.** Summary of key findings

Honeycomb category	Key findings
Findability	Not assessed
Understandability	Technical jargon should be kept to the minimum amount possible The resource should be explained by a healthcare professional Diagrams should be kept as simple as possible Technical and numerical information should be kept as simple as possible Women could find it helpful to be guided through the resource in discussion with a HCP^I^ to aid understanding
Usefulness	Content that was particularly useful included information about the different operations that use mesh and what patients should do if they feel worried about complications Content that was not considered to be useful included signposting to GP^II^ services and the suggestion that patients can access their own medical notes was also viewed as inappropriate or unrealistic Signposting to support/community groups and nurse-led services would be useful
Usability	Bullet points break up the text and are therefore a useful way to present information A large, bold font should be used for titles and headings Keep the volume of information to the minimum possible Diagrams must be clearly labelled and well positioned on the page An A5 booklet style is preferable Having a trusted health professional to co- navigate and highlight aspects of the leaflet was seen as potentially valuable
Desirability	Resources for the public should be eye catching and appealing Patient information can be ‘scary’ for readers, which can be off-putting for some
Credibility	Using branding (such as trusted NHS^III^ logos) can make a resource appear credible An honest tone can help manage patients’ expectations
Affiliation	Try to use simple language, and define medical terminology, appropriate for the public
Accessibility	Reducing the reading age to the lowest possible level will increase accessibility, although this is challenging because technical medical information must be included in patient resources

^I^Healthcare professional

^II^General practitioner

^III^National Health Service

Through the iterative analysis process, it became clear that the facets of the user experience are closely inter-related. For example, a barrier to usability will often impact understandability as a result. In addition, there were a number of themes that were found to run across several categories. For example, the use of technical jargon was a theme captured by both the understandability and affiliation categories.

### Understandability

The principal theme under understandability was clarity and the ease of understanding the language. While some participants felt the language was clear and easy to understand, others felt it was overly technical with too much jargon. Terminology highlighted as particularly confusing, included *sacrocolpopexy and inert*.

Participants also described difficulty understanding the difference between *symptoms* and *problems* and suggested that the explanation of *Retropubic Transvaginal Tape* was overly detailed. Several participants suggested that there was too much information and that this affected the understandability of the resource. Some participants stated that they would have to read parts of the resource more than once to be able to understand it.

I would really have to read that again….I think it’s the 4th bullet point down, ‘the mesh material is inert’. I’ve no idea what that means… (Kate).

A number of participants also suggested that the resource should be explained by a healthcare professional (HCP), to ensure that patients and families properly understand the information.

I think it’s quite detailed… I think it might be quite difficult for some people to understand it, so I think it would probably something that it would be better that somebody took the patient through the leaflet rather than just giving them it away to read…. there is quite a lot of technical language in it and it might need explaining (Ingrid).

**Fig. 1:**
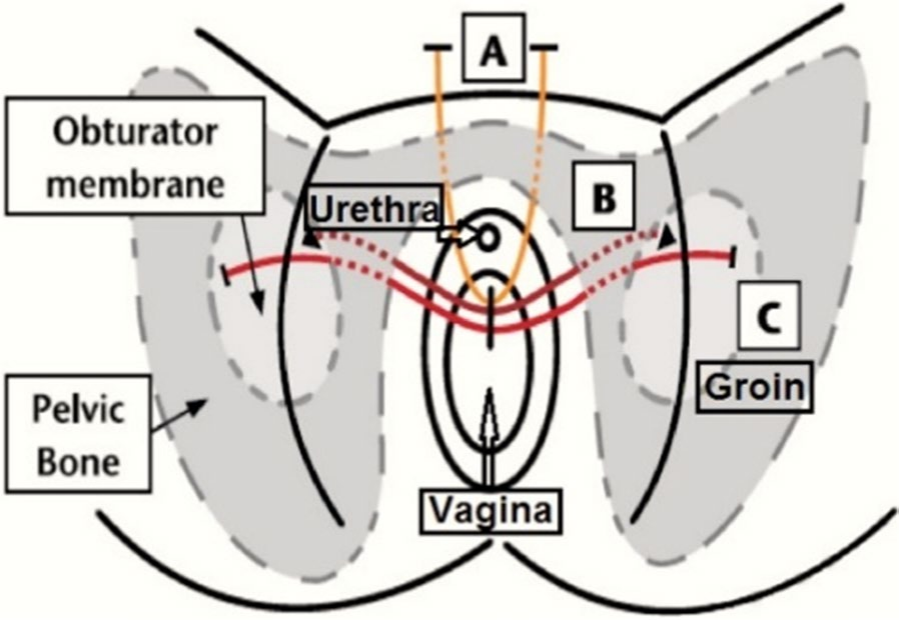
Diagram taken from the Vaginally Inserted Synthetic Mesh: Potential Complications resource. The diagram in the complications resource was developed by Dr Julia Wilkens, subspecialist in Urogynaecologist, NHS Lothian, who developed the whole resource. The author of the leaflet has confirmed that a copyright permission is not necessary to reproduce the figure due to it being designed for free distribution

A number of participants highlighted the value of including a diagram to facilitate understanding of patient information. However, with the exception of two participants who liked the diagram (Fig. 1) [see Additional file 1], the other participants agreed that the diagram was confusing because of the technical detail, labelling and/or the position of the diagram on the page. These reasons highlight the interplay of understanding and usability.

“I’m not so sure about the diagram, being of any use to anybody that’s not a gynaecologist (Claudia).

**Fig. 2:**
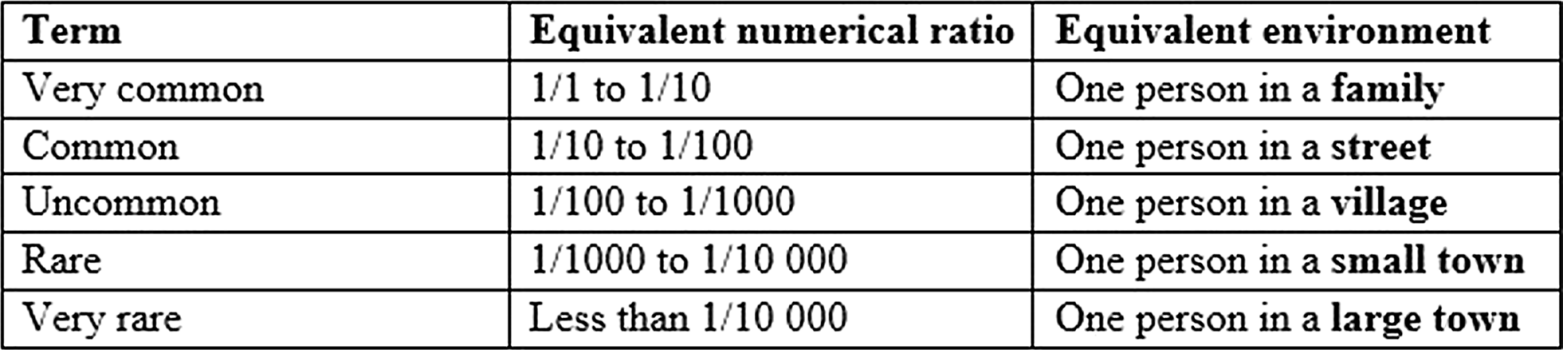
Table from the *Vaginally Inserted Synthetic Mesh: Potential Complications* resource

Some participants expressed strong opposition to the table presented in the complications leaflet (Fig. 2) [see Additional file 1] describing the rate of potential complications, explaining that it caused confusion and/or was unnecessary. A small majority of participants, however, explained that the table promoted understanding by putting the rate of complications into perspective and, therefore, the table was included in the final version of the complications leaflet.

### Usefulness

In general, participants described the resource as “informative” and considered each section helpful. Participants acknowledged a lack of patient resources for the complications of mesh implants and welcomed the resource as an alternative to online information, which they described as overwhelming. Several participants also highlighted that carers and family members may also find the resource useful.

Three key themes emerged from this category: information that is useful, information that is not useful and information that would be useful. Particularly useful content related to the risks and outcomes of mesh removal and what patients should do if they are worried about their implant. Some participants also commented that information about the different types of operation that use mesh implants (for example, retropubic and trans-obturator procedures) was useful. Conversely, other participants reported that the volume of information and level of detail describing these operations was unhelpful and off-putting. Based on their experiences, some participants also reported that signposting to GP services and the multidisciplinary team was either unrealistic or not the appropriate pathway of care. Similarly, some participants disagreed with advice in the resource that suggests patients might be able to access their own medical records, describing this content as unrealistic and therefore unhelpful.

I don’t think people should be going to their GP when they’ve had major surgery… they should be going straight back to their consultant…the GPs are quite busy at the moment, so maybe they are not going to get seen as quickly as they should, maybe an infection could grow… That’s why it might be useful if there could be something like a mediation between the consultant and the patient, like a helpline … (Emma).

…does it need to say all that – about what type of mesh you have…First wee bit [the first little bit] is fine but then it goes on to say about your operation notes. Like how easy is it to get access to your own notes, that bit is nonsense. (Louise).

Additional information that would be useful to include in the resource, is signposting to support groups and nurse-led services, which participants described as valuable services.

### Usability

The user-friendly format was a key theme to emerge from this category. Overall participants liked the font size and style. Participants considered bullet points a good way to break up the text and underlined text helped draw attention to important information. The larger size of the headings and the bold font used for the sub-headings also made it easier to read and navigate the document. Nonetheless, several participants still suggested that, given the volume of information, HCPs could play an important role in helping patients navigate the information that is most useful/relevant to them.

…I’d expect him [GP] to go through this and say this bit is what I think refers to you. Not just say, there you go, go away and read it. I think he needs to take a little bit of time to say….you’ve got mesh exposed in your vagina and if you look this section, this explains to you about that. (Hillary).

Participants offered three key suggestions to enhance usability. Firstly, a number of participants experienced difficulty referring to the diagram due to its position, which made participants flip back and forth through the pages. To solve this problem, participants suggested moving the diagram adjacent to the text that refers to it. Secondly, although the title was described as eye-catching, some participants suggested that the ‘*potential complications*’ part of the title should either be a larger font or highlighted with colour (see Additional file 1, to make it clear exactly what the resource is about. A number of participants also suggested that the resource would be more usable in an A5 booklet form, which could also improve desirability.

### Desirability

Most participants indicated that they would not be drawn to pick up the resource because they did not consider it to be physically appealing. One participant stated that it looked like an “office document” and another described it as “boring”. Despite its physical appearance, a number of the participants reported that they would pick up the resource because of personal experience with mesh implants or because they were interested due to the media coverage that mesh implants had received.

I probably would [pick it up] as I am curious about it especially with all press there’s been about mesh… (Fiona).

Desirability also manifested in the emotional reaction of the majority of participants, who frequently described the content as “scary”, “frightening” or “threatening”. Such reaction was expressed by both patients and staff.

But reading it, I’m kinda [sort of] shaking and a bit taken aback by it, em it’s actually quite horrific (Kate).

However, this negative emotional reaction did not impact the desirability of the resource and a number of participants acknowledged that patients experiencing problems might find the level of detail contained in the resource reassuring and useful.

I think that’s good. Very wordy. Although I suppose if you were badly affected you would probably want to read more, you would probably take comfort from the amount of information that’s being given. (Hillary).

### Affiliation

Affiliation describes how a user identifies with a resource (for example, whether users feel that the resource is for someone like them). It was incorporated by Rosenbaum to capture aspects of the user experience relating to identification, membership and alienation [15].

Insights on affiliation illustrate that the experience of using the resource was similar for both patients and HIS staff, regardless of their educational background. Although none of the participants expressed feelings of alienation, one participant suggested that older users might struggle to understand the content and several others felt that the technical language and the level of detail was targeted towards users with existing medical knowledge (such as a *gynaecologist, nurse* or *medical student*), rather than a lay person. Nonetheless, a number of participants acknowledged that patients with experience of mesh are likely to have a background level of understanding, which might make it easier for them to understand the material.

It’s certainly very very thorough, I would say, to some of these it’s more appropriate for a medical student, it covers such detail. (Daisy).

I think it is clear. Clearer to people going through it, as they would understand the words. (Jessica).

### Credibility

Overall, participants considered the resource as trustworthy. Participants agreed that they would take its advice because the NHS branding indicated that it was from a reputable source. One participant reported that she would trust the information in the resource if it was given to them by a doctor, further emphasising the important role of the HCP in the dissemination of the resource.

Yes definitely [I would use the advice in this leaflet]. It looks well established, supported with logos and stuff. (Emma).

In addition, some participants commented that, even though some of the content was “frightening”, the level of detail, clarity and openness about factual information was actually useful and credible:

I don’t think there is anything I would remove…. because I feel that that’s information that you would need to know, I think it’s being transparent and being open…… Quite harrowing reading it, but very clear and no false promises (Kate).

However, a few participants disputed the accuracy and appropriateness of some of the information relating to mesh implants and autoimmune disease, based on their understanding of the evidence or their personal experiences with mesh implants.

I know that there’s a lot of that, that one wee bit [little bit], in telling you there’s no evidence to support, but it’s no telling if there’s any evidence that refutes it (Claudia).

### Accessibility

In describing accessibility, Morville [20] writes:

Just as our buildings have elevators and ramps, our web sites should be accessible to people with disabilities.

Given that the resource was presented to participants as a hard copy, physical accessibility was not directly assessed. However, a few participants commented on the benefit of having a paper resource disseminated by a medical professional (discussed under credibility) as opposed to searching for information online, which could be overwhelming. Conversely, a couple of participants indicated that they would prefer to use the resource in an online format.

Although not discussed by participants directly, concerns about the technical language, illustrations used, and volume of information identified earlier indicate that the resource may not be suitable for members of the public who have a low reading age (the level of reading ability of a person which is comparable to that of a child) or low health literacy.

## Discussion

The user testing interviews captured important information on the content, language, presentation and dissemination of the *Vaginally Inserted Synthetic Mesh: Potential Complications* resource. Detailed feedback from this review regarding facilitators and barriers, as well as suggested improvements, were shared with the clinician responsible for developing the resource and several revised iterations and improvements were made, building on the findings of this study. This included as a headline message that women might find it helpful to share discussions and talk through the leaflet with a HCP. An executive decision to arrive at a final version of the complications leaflet [see Additional file 2] was made by representatives from NHS Lothian. Some of the suggestions made by the study were not possible to implement as there were different opinions among NHS Lothian representatives. By the time of writing this article, the revised material was due for wider circulation across Scotland.

Key facilitators typically promoted usability and understandability, including the use of a font (size and style) that is easy to read, bullet lists, bold headings and simple language. The use of NHS branding was also highlighted by participants as a facilitator that promoted credibility.

However, the use of technical/clinical language (particularly the terms *sacrocolpopexy and inert*) was a key barrier to understandability and could affect accessibility for users with a low reading age. Similarly, health information that is difficult to understand presents a hurdle to those with low health literacy. Health literacy is the ability to obtain, understand and use health information to actively participate in decision-making and navigate health services [21]. The findings from our study are in line with previous research which highlights the impact of limited health literacy on the publics’ ability to independently use a decision aid [22] and to make good decisions about their health [21]. The finding suggests that the information in the resource should be appropriately designed to include the use of plain language and avoid the use of technical jargon in order to be usable by all audiences.

The volume of information and the use of the technical diagram were also considered by the majority of participants as barriers to the understanding of the patient leaflet on complications of mesh implants. Some participants, however, considered them as beneficial for understanding of patient information. Opinion was also divided over the terms describing the rate of potential complications, although in this case more participants felt it aided their understanding than those who felt otherwise. These examples illustrate the challenge of creating a resource for a diverse target audience with a range of knowledge and experience. This is a finding that has been reported consistently from research conducted as part of work package three of the DECIDE project, which aimed to improve the international practice of disseminating evidence-based recommendations and develop strategies for producing and disseminating patient versions of clinical guidelines [23]. Some of the key findings from the DECIDE project, relevant also for the leaflet on complications of mesh implants, were that numerical summaries of data can be useful if they allowed users to interact with them so that they can choose the level of detail that they require and that information presented in patient versions should move beyond accuracy and precision and start talking about the effect on important patient outcomes.

Presenting information in a layered format may be one solution to balancing the information needs of a varied audience. According to the Guidelines International Network Public toolkit, layering content involves careful consideration of the order of information and presentation of the most important information first. In online resources, layering can be achieved using drop-down menus and hyperlinks, which can enable the user to select the appropriate level of detail for their needs [24]. In hard-copy materials, information can be layered by the use of a clear contents page that facilitates flicking to the desired sections, creative use of formatting (for example, use of boxes, table and visuals) and careful consideration of the order of presentation of information [16].

For the most part, feedback regarding the facilitators and barriers to understanding the mesh implant patient information leaflet is consistent with existing research, adding to the growing literature about best practice principles for writing patient information. Some of these best practice principles include making complex information easy to use and to understand, the use of appropriate typography (for example, the use of columns and white space within the written text), use of headings and sub-headings on the page to help the reader navigate the information, and use of colour, symbols and pictograms to aid understanding of information [25]. Importantly, through sharing their experience of accessing health services, participants identified content that did not reflect their experiences (for example, accessing medical notes) and additional sign-posting opportunities (for example, to nurse-led services).

The role of the HCP was also an important theme that emerged from this user testing. Participants suggested that by disseminating the resource, HCPs could promote trust and understanding, as well as help users navigate the relevant content of the mesh implant patient information leaflet. The role of health professionals in aiding health literacy was also underscored in the feedback. Given the adverse emotional reaction to some of the content, an additional advantage might be that HCPs can offer support to users that find the content frightening or distressing.

Although HCPs can play an important role in disseminating the resource, a wealth of literature demonstrates that people are increasingly looking to the internet for health information [26, 27]. Online health information can help reduce anxiety and empower patients in their decision-making by improving understanding and providing reassurance or a second opinion [26]. If published online, the complications resource for mesh implant could prove to be a valuable source of credible patient information, especially given the dearth of high quality patient information relating to mesh complications [28, 29]. However, to promote a positive user experience in an online environment, it is necessary to assess findability of the resource and to address issues relating to understandability, such as fine-tuning the information elements and layout to enable easy navigation. The plan at the time of writing this paper was to share the leaflet with NHSScotland and make it freely available on the NHS Inform website. The leaflet is intended for women who have already undergone a mesh procedure for SUI or POP.

### Strengths and limitations

A strength of this study lies in the use of an established and credible method of user testing and a validated model of user experience. This method of user testing has proven effective in enabling patients and information providers to judge the quality of written information about treatment choices in a systematic and structured way [30]. Previous research has also used the DISCERN tool to assess patient information related to SUI and POP [16]. A further strength of the study is the involvement of multiple analysts in the data analysis which provided a check on selective perception and a rich data analysis that may not otherwise be achieved with a single researcher [31].

The extent to which these findings are transferrable, however, may be limited due to the method of sampling. Although recruiting a convenience sample of HIS staff increased the heterogeneity of the sample (with regard to age and level of education), it is possible that these participants had a greater knowledge of mesh implants than the general public which could have impacted the way they understood the information that was presented in the complications leaflet. Furthermore, although there were no variations in user experience by level of education, this study did not assess participants’ health literacy level or reading age. Given that all participants were women residing in Scotland, the findings may not reflect the experiences of other potential users, men or family members/carers, and it is not possible to draw conclusions about the cultural acceptability of the resource.

## Conclusion

The findings strongly illustrate the value of undertaking in-depth user testing for patient information resources before their dissemination. In this study the senior clinician involved in developing the patient information leaflet on complications of mesh implants supported and welcomed a user testing stage. The findings brought new insights not just about the need for clear accessible content and visual presentation of the information but also highlighted how the direct guidance or navigation of a patient information resource by a HCP could increase its salience and accuracy of interpretation. The interviewees highlighted the importance of being able to ask for clarification of any complex terms and seek reassurance about any fears. This shared approach between user and professional could enable informed decision-making.

The study also reinforces the need to assess contextual factors when developing patient information resources. The mesh complications leaflet was developed in a context of heightened public and political concern in Scotland (and more globally) about the efficacy of mesh treatments. In such an environment with high media interest in vaginal mesh implant procedures, the role of a supportive HCP as part of the communication process could improve understanding, customise messages, correct any misinformation and create a receptive environment for the patient information resource.

This study raises wider implications in terms of pointing to the need to allow realistic evaluation time for the collation of patient input and wider stakeholder views before any patient information material is disseminated. The qualitative approach with interviews and direct observation in this study yielded rich insights and reflections. It is time–intensive but worthwhile in terms of the quality and range of insights gleaned. As acknowledged above, it brings limitations in terms of wider generalisability. Future development of complex or sensitive patient information resources could build on this original work and scale up and complement in depth exploration with survey approaches.

## Supplementary Information

**Additional file 1**. Vaginally Inserted Synthetic Mesh: Potential Complications resource - before user testing.

**Additional file 2**. Vaginally Inserted Synthetic Mesh: Potential Complications resource - final version.

## Data Availability

The datasets generated and/or analysed during the current study are not publicly available due to a risk of individual privacy being compromised. The datasets are available from the corresponding author on reasonable request.

## Appendix 1

**Table:**

1	**Present with Vaginally inserted synthetic mesh potential complications information leaflet for patients and carers** Please have a look and tell me what you think of it
1a	**What are your first impressions?**
1b	**If you saw this would you pick it up? What do you think of the way it looks?**
1c	**Do you know who this is for?**
1d	**How do you find the title?**
2	**Present with introduction page only** Please have a look at just the introduction and tell me what you think of it
2a	**Does this tell you what you want to know about whether the leaflet is for you?**
2b	**Is it clear?**
3	**Present with **‘**why are synthetic meshes inserted into the vagina?**’ Please have a look and tell me what you think of these pages
3a	**Is this information clear?**
3b	**Is it helpful?**
3c	**Would you add or remove anything?**
4	**Present with **‘**what are the problems I could have after prolapse or incontinence surgery?”** Please have a look and tell me what you think of these pages
4a	**Is this information clear?**
4b	**Is it helpful?**
5	**Present with ‘what are the problems that the mesh could be causing?**’ Please have a look and tell me what you think of these pages
5a	**Is this information clear?**
5b	**Is it helpful?**
6	**Present with ‘What do I do if I am worried?**’ Please have a look and tell me what you think of these pages
6a	**Is this information clear?**
6b	**Is it helpful?**
7	**Present with ‘Do I need to have the mesh removed?**’ Please have a look and tell me what you think of these pages
7a	**Is this information clear?**
7b	**Is it helpful?**
8	**Present with ‘what are the risks of mesh removal?**’ Please have a look and tell me what you think of these pages
8a	**Is this information clear?**
8b	**Is it helpful?**
9	**Think about the leaflet as a whole. Have another look through it if you want**
9a	**Does it look appealing? Would you pick one up?**
9b	**Would you add anything to or remove any of the information?**
9c	**How do you find the language in it? Would you change it at all?**
9d	**Would you use the advice and information in this leaflet? Why?**
16e	**Is there anything missing from it that you would like to know about?**
16d	**Is there anything you would changes about the leaflet?**
17	**Thank you very much – that**’**s all. Anything else to add**

## Abbreviations

SUI: Stress urinary incontinence
POP: Pelvic organ prolapse
HIS: Healthcare Improvement Scotland
TVMO: Transvaginal mesh implant oversight group
HCP: Healthcare professional

## References

[1] Pakbaz M, Persson M, Löfgren M, Mogren I (2010). 'A hidden disorder until the pieces fall into place' - a qualitative study of vaginal prolapse. BMC Womens Health.

[2] National Institute for Health and Care Excellence. Urinary incontinence and pelvic organ prolapse in women: management. 2019. 31211537

[3] Information Services Division. Transvaginal Services in Scotland. 2019. https://www.isdscotland.org/Health-Topics/Hospital-Care/Publications/2019-10-08/2019-10-08-Transvaginal-Mesh-Procedure-Summary.pdf

[4] Morling JR, Mcallister DA, Agur W, Fischbacher CM, Glazener CMA, Guerrero K (2017). Adverse events after first, single, mesh and non-mesh surgical procedures for stress urinary incontinence and pelvic organ Prolapse in Scotland, 1997–2016: a population-based cohort study. Lancet.

[5] Scottish Government. Scottish Independent Review of the use, safety and efficacy of transvaginal mesh implants in the treatment of stress urinary incontinence and pelvic organ prolapse in women: final report. 2017. https://www.gov.scot/publications/scottish-independent-review-use-safety-efficacy-transvaginal-mesh-implants-treatment-9781786528711/. Accessed 16 Mar 2020.

[6] Medsafe. Surgical Mesh Implants. Regulatory action on surgical mesh products. 2018, https://www.medsafe.govt.nz/hot/alerts/UrogynaecologicaSurgicalMeshImplants.asp. Accessed 16 Mar 2020. https://www.medsafe.govt.nz/hot/alerts/UrogynaecologicaSurgicalMeshImplants.asp. Accessed 16 Mar 2020.

[7] House of Commons Hansard. Medicines and Medical Devices Safety Review. 2018. https://hansard.parliament.uk/Commons/2018-02-21/debates/7DA2E2F3-E1E6-40CB-8061-680E0399CA97/MedicinesAndMedicalDevicesSafetyReview. 16 Mar 2020.

[8] NHS Improvement and NHS England. Letter from Prof Stephen Powis and Dr Kathy McLean to Acute trust CEOs and medical directors. 2018. https://i.emlfiles4.com/cmpdoc/9/7/2/8/1/1/files/47633_mesh-letter-to-acute-ceos-and-mds.pdf Accessed 16 Mar 2020.

[9] Scottish Government. Halt in use of transvaginal mesh. 2018. https://www.gov.scot/news/halt-in-use-of-transvaginal-mesh. Accessed 16 Mar 2020.

[10] Healthcare Improvement Scotland. Transvaginal Mesh Implants Oversight Group (TVMO) - Flash report. 2019. http://www.healthcareimprovementscotland.org/our_work/technologies_and_medicines/programme_resources/idoc.ashx?docid=f82922b3-3a97-4c61-b109-164a4edae624&version=-1. Accessed 10 Jan 2020.

[11] NHS Lothian. Vaginally Inserted Synthetic Mesh: Potential Complications. Version 1. 2019.

[12] Maat HP, Lentz L (2010). Improving the Usability of Patient Information Leaflets Patient Education and Counseling. Patient Edu Couns.

[13] Sonal SM, Unnikrishnan M, Vyas N, Rodrigues GS (2017). Development and evaluation of patient information leaflet for diabetic foot ulcer patients. Int J Endocrinol Metab.

[14] Treweek S, Oxman AD, Alderson P, Bossuyt PM, Brandt L, Brożek J (2013). Developing and evaluating communication strategies to support informed decisions and practice based on evidence (DECIDE): protocol and preliminary results. Implement Sci.

[15] Scottish Intercollegiate Guidelines Network. SIGN 144: Glaucoma referral and safe discharge. 2015. https://www.sign.ac.uk/assets/pat144.pdf%20Accessed%2016%20Mar%202020. Accessed 12 Jan 2020.

[16] Fearns N, Graham K, Johnston G, Service D (2016). Improving the user experience of patient versions of clinical guidelines: user testing of a Scottish Intercollegiate Guideline Network (SIGN) patient version. BMC Health Serv Res.

[17] Rosenbaum SE, Glenton C, Cracknell J (2008). User experiences of evidence-based online resources for health professionals: user testing of The Cochrane Library. BMC Med Info Dec Making.

[18] Phillippi J, Lauderdale J (2017). A guide to field notes for qualitative research: context and conversation. Qual Health Res.

[19] Jokanovic NAP, Carter S (2019). Development of consumer information leaflets for deprescribing in older hospital inpatients: a mixedmethods study. BMJ Open..

[20] Morville P. User Experience Design. 2004. http://semanticstudios.com/user_experience_design/. Accessed 12 April 2019.

[21] Scottish Government. Making it easy: A health literacy plan for Scotland. 2014. https://www.gov.scot. Accessed 10 Jan 2020.

[22] Scalia P, Durand M, Faber M, Kremer J, Song J, Elwyn G (2019). User-testing an interactive option grid decision aid for prostate cancer screening: Lessons to improve usability. BMJ Open.

[23] Developing and Evaluating Communication Strategies to support Informed Decision and practice based on Evidence (DECIDE). DECIDE 2011–2015. https://www.decide-collaboration.eu/sites/www.decide-collaboration.eu/files/public/uploads/DECIDE%20public.pdf Accessed 10 Jan 2020.

[24] Graham K, Treweek S, Santesso N, Schaefer C. How to develop patient versions of guidelines. In: G-I-N Public Toolkit: Patient and Public Involvement in Guidelines. 2015. https://g-i-n.net/document-store/working-groups-documents/g-i-n-public/toolkit/toolkit-2015/view Accessed 10 Jan 2020.

[25] MHRA MaHpRA. Best Practice Guidance on Patient Information Leaflets. 2014.

[26] Dutton WH, Blank G, Groselj D. Cultures of the internet: The internet in Britain. Oxford Internet Institute; 2013. http://oxis.oii.ox.ac.uk/. Accessed 10 Jan 2020.

[27] Rowley J, Johnson F, Sbaffi L (2017). Gender as an influencer of online health information-seeking and evaluation behavior. J Assoc Info Sci Tech.

[28] Eysenbach G, Wyke S, Powell J, Inglis N, Ronnie J, Large S (2011). The characteristics and motivations of online health information seekers: cross-sectional survey and qualitative interview study. J Med Internet Res.

[29] Duenas-Garcia O, Kandadai P, Flynn M, Patterson D, Saini J, O'Dell K (2015). Patient-focused websites related to stress urinary incontinence and pelvic organ prolapse: A DISCERN quality analysis. Int Urogyn J.

[30] Charnock DS, Needham G, Gann R (1999). DISCERN: an instrument for judging the quality of written consumer health information on treatment choices. J Epidemiol Commun Health.

[31] Patton M (1999). Enhancing the quality and credibility of qualitative analysis. Health Serv Res.

