# Supplementary material for: User testing a patient information resource about potential complications of vaginally inserted synthetic mesh
Source: BMC Womens Health. 2021 Jan 25;21:35. doi: 10.1186/s12905-020-01166-4 (PMC7831188; doi:10.1186/s12905-020-01166-4)
Supplement: Supplementary file 2 — Additional file 2. Vaginally Inserted Synthetic Mesh: Potential Complications resource - final version. [file 12905_2020_1166_MOESM2_ESM.pdf]

NHS Lothian

Vaginally Inserted Synthetic Mesh

Potential Complications

Patient Information Leaflet

Who is this leaflet for?

This leaflet is for you if you have previously had a synthetic mesh inserted into the vagina, and you are concerned that this may be causing problems.

You may find it helpful to go through the leaflet together with a health professional.

Surgeons use synthetic mesh for different procedures to help with urinary stress incontinence (SUI) or pelvic organ prolapse (POP). The type and amount of mesh material used and the way it is placed may vary significantly. It is therefore very important to know the exact details of your procedure if you want to find out whether any of your symptoms are related to the mesh that has been inserted. These details will be recorded on your operation notes. If you wish to have more information about your surgery, your GP may be able to help you or refer you to the surgeon who performed your operation.

Why are synthetic meshes inserted into the vagina?

Synthetic meshes are used to cure or improve urinary stress incontinence (SUI) or pelvic organ prolapse (POP).

To help with incontinence, surgeons place synthetic mesh tape like a sling under the urethra (the tube coming out of your bladder) to support it. You may have had a retropubic TVT (tension free vaginal mesh tape) procedure, which means you had a small cut in your vagina and two small cuts just above your pubic bone (Figure 1A in yellow). Alternatively, you may have had a trans-obturator TVT-O/TOT (tension-free vaginal trans-obturator mesh tape) procedure with a small cut in your vagina and two small cuts, one in each side of your groin (Figure 1C, in red). There are also some tapes that are put in through a single incision in your vagina, and they would have been fixed with little anchors into a membrane, called the obturator membrane, that is part of your pelvic bone (Figure 1B, in dark red). Please see the diagram below.

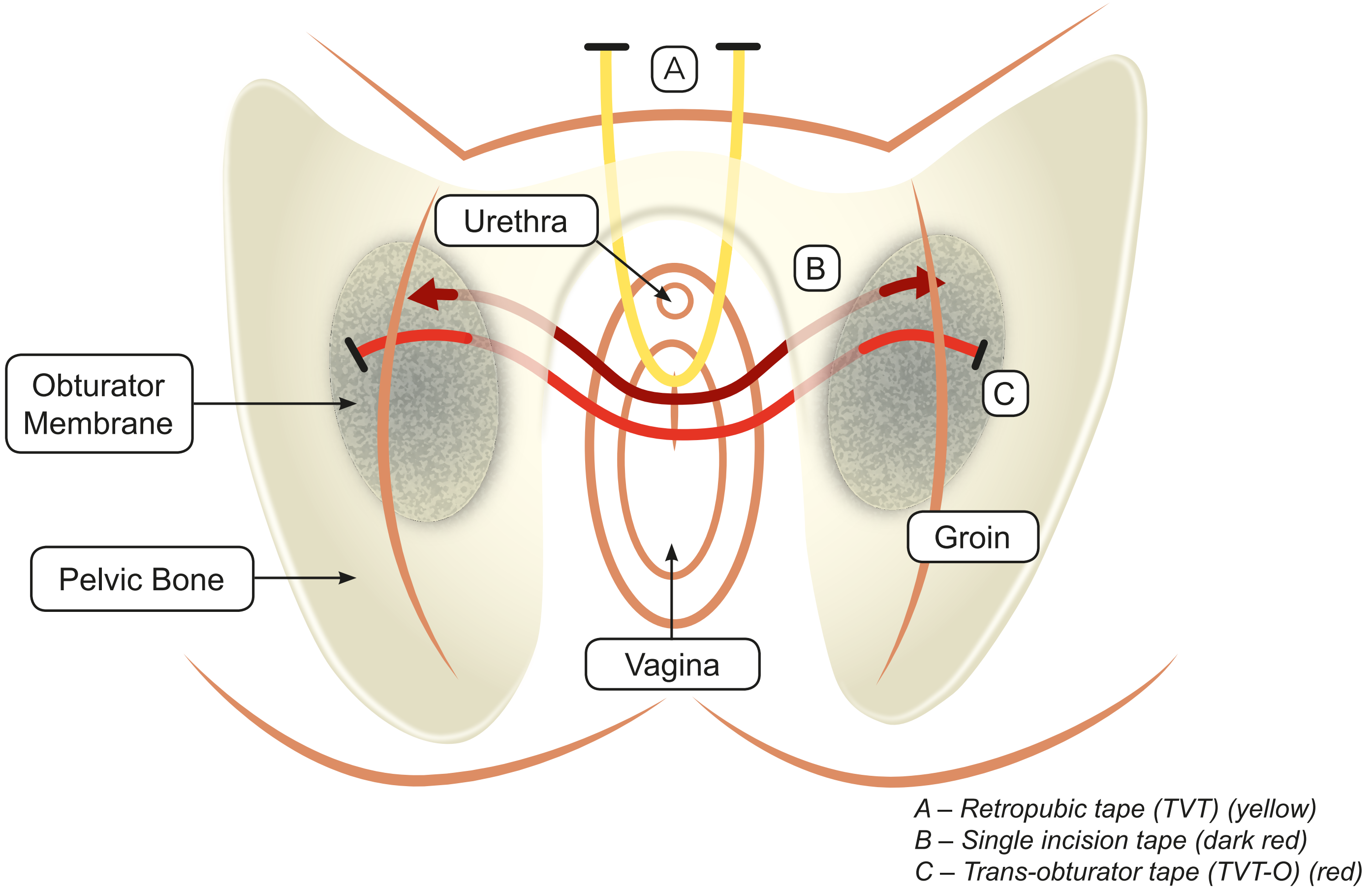

Figure 1 – Tension-free vaginal tapes to treat stress urinary incontinence

Vaginal meshes for prolapse (POP) are usually bigger pieces that support the vaginal walls as part of a pelvic floor repair. There is also a procedure called “sacro-colpo-pexy” to treat a prolapse through the abdomen, where a piece of mesh is attached to the top of the vagina or the neck of the womb to hold it up. Although synthetic mesh is used for this, it is a very different procedure and does not involve insertion of mesh through your vagina.

The mesh material used for all these different procedures is the same. It is also the same mesh that’s used by surgeons to repair hernias.

What are the problems I could have after prolapse or incontinence surgery?

There are a number of potential complications following operations for incontinence or prolapse, **whether or not any mesh was used.**

The terms used to help you understand how likely it is for these complications to happen are explained in the table below:

Term	Equivalent numerical ratio	Equivalent environment
Very common	1/1 to 1/10	One person in a **family**
Common	1/10 to 1/100	One person in a **street**
Uncommon	1/100 to 1/1000	One person in a **village**
Rare	1/1000 to 1/10 000	One person in a **small town**
Very rare	Less than 1/10 000	One person in a **large town**

Potential complications:

- **Difficulty passing urine:** Sometimes an operation for incontinence or prolapse affects the way the bladder is working and you may find it difficult or impossible to empty your bladder. This is very common especially if you had an operation for incontinence. It may only take a few days to settle and you just need to have the catheter for a bit longer. It is common that it may take a few weeks to get back to normal. If that happens, you are shown how to use a little tube to empty your bladder yourself. It is uncommon for this to become a long term problem.

- **Irritable bladder symptoms:** The main purpose of stress incontinence surgery is to treat urinary leakage which happens during activity, coughing, sneezing, laughing etc. It is not expected to help with irritable bladder symptoms, such as needing to pass urine often and having trouble getting to the toilet in time. It is a very common risk that irritable bladder symptoms develop or get worse after surgery for stress incontinence (in up to 15% of cases).

- **Injury to bladder / urethra / bowel:** Operations for incontinence or prolapse are done through the vagina. The bladder, the tubes going in and out (ureters and urethra) and the bowel are all very close, and so there is a risk of injury to these structures during vaginal surgery. If the injury is noticed and repaired at the time, long-term problems are unlikely. If any injury happened during your operation, your surgeon should have told you about this at the time of your procedure.

- **Pain / Numbness in the scar area:** Normally, operations cause pain, but this tends to settle during the recovery period. It is uncommon for the pain to last longer and take more time to settle. If the pain lasts longer, this may be due to irritation of nerves or due to scar tissue, which could then become a long-term issue. It may also happen that irritation of nerves causes a change or loss in sensation or numbness in the area of the scars.

- **Discomfort with sexual intercourse:** Operations in the vagina change the shape of the vagina, and there may also be some scarring. The vagina may not stretch very well or be narrower than before, and then sexual intercourse may cause some discomfort or pain. This usually settles as the tissues heal but may become a long-term problem.

- **Recurrence or persistence of symptoms:** With any surgery, there is the risk that it doesn't work as well as expected, and your symptoms may either not completely go away or come back after some time.

What are the problems that the mesh could be causing?

Synthetic mesh in the vagina acts to reinforce your own tissues in both incontinence and prolapse procedures. It will also lead to scar tissue forming around the mesh which will help further with the support.

- Problems may be caused if the mesh becomes exposed or gets very close to the surface of the vagina or the bladder. This is a common risk. It may be exposed soon after you had it put in, but it is also possible that the mesh gets exposed into the vagina or the bladder months or even years after your surgery. If the mesh is exposed in the vagina, there is a risk that the mesh may become infected.

- It may happen that the mesh is placed very close to other structures, such as your pelvic bone or some of your nerves. This may then cause pain, and typically you would have pain immediately after your operation.

- There may be too much tension on the mesh. If you had a mesh tape to treat incontinence, you may find it difficult or impossible to pass urine. You may also experience pain, especially with intercourse. On the other hand, if the mesh is too loose, it may not work.

- The mesh material is inert, which means that it is unlikely to cause any reaction. There is currently no good evidence to support a link between mesh and autoimmune diseases. The mesh that has been inserted into your vagina is very unlikely to cause any symptoms outside the area where it has been placed (pelvis / groin). The mesh material does not break up or dissolve and it does not spread to any other parts of your body. There is also currently no evidence that the mesh material releases any chemicals or toxins that would harm you in any way.

Noted below are the symptoms you may have if the mesh is causing problems:

Mesh exposed in the vagina

Mesh exposure in the vagina may happen if the vaginal skin does not heal properly at the time of your surgery. It can also happen when the mesh works its way through the vaginal skin over time. It may then be possible to feel the mesh coming through the vaginal skin when you are examined.

- **Bleeding:** You may get some bleeding from your vagina. The bleeding may happen anytime or specifically after intercourse or strenuous activities.

- **Vaginal discharge:** If the mesh is exposed in the vagina, there is a high risk that it becomes infected, which will then cause a smelly discharge.

- **Problems with sexual intercourse:** Mesh exposed in the vagina is likely to cause pain with intercourse, both for you and for your partner. It may cause a small injury to your partner due to the rough exposed surface of the mesh.

Mesh exposed in the bladder, ureters or urethra.

Mesh exposure in the bladder, ureters or urethra may happen at the time of the surgery or because the mesh works its way through over time. At the time of your operation, your surgeon would normally have looked into your bladder with a camera (cystoscopy) to make sure that no mesh was exposed.

- **Blood in your urine:** The mesh may cause some bleeding and there may be blood in your urine, either for you to see or when your urine is tested.

- **Recurrent urine infections:** The exposed mesh may become infected and cause you to have recurrent urine infections. If the mesh is exposed for any length of time, a stone may form in your bladder / urethra, which also increases the risk of infections.

- **Irritable bladder symptoms:** Exposed mesh may irritate your bladder and cause you to go to the toilet more often or to have to rush to the toilet.

- **Difficulty emptying your bladder:** If the mesh is exposed in your urethra, it may stop the urine from flowing properly.

Symptoms due to tension or irritation by the mesh

f the mesh is causing any pain, the pain will be located in the area where the mesh was placed. It may be helpful for you to be examined by a specialist to see where exactly you are feeling pain and to find out if it may be related to your mesh.

Increased tension on the mesh may cause pain, pain during sex and possibly bladder problems (such as bladder irritability or difficulties with bladder emptying).

What do I do if I am worried?

If you are experiencing any symptoms that you think may be related to mesh, you should see your GP. Your GP may refer you back to the surgeon who did your mesh procedure for assessment of your symptoms or alternatively to the urogynaecology or urology department.

The detailed information about the exact procedure on your operation note together with your present symptoms will be assessed by the specialist service to which you may be referred, to consider what treatment may be appropriate for you.

Do I need to have the mesh removed?

- If your mesh is infected, as much of it as possible will need to be removed. It will not be possible to completely clear the infection without removing the infected mesh.

- If your mesh is exposed into your bladder, ureters, urethra or bowel, at least the exposed portion of the mesh or the complete mesh will need to be removed. Your symptoms are not likely to get better and may get worse, if the exposed mesh is left in place.

- If the mesh is exposed in your vagina, the exposed portion of the mesh will most likely need to be removed. It may be possible to use the vaginal skin to cover up the exposed mesh. If the exposed portion is very small, the vagina may heal with the use of hormone (estrogen) cream, but if it has not healed after a few weeks, the exposed mesh should be removed.

- If it appears that the mesh is too tight, it may be possible to release the tension by cutting it without having to remove it. Sometimes this is enough to help with your symptoms. Cutting the mesh is a much smaller procedure than removing it.

- If the mesh does not appear to be either too tight or exposed, removing it may not have any benefits for you. If you are troubled by pain and especially if you developed pain sometime after your initial operation, it is important to know that removing the mesh may not improve or even worsen the pain.

- Any symptoms outside of your pelvic or groin area (for example, general fatigue / blurred vision / nausea / constipation / itchy skin) will not be expected to change with removal of your mesh.

What are the risks of mesh removal?

Any surgical procedure has risks and potential complications, which need to be carefully considered and weighed against the expected benefits. This is true for procedures using mesh but also for surgery to remove it. The more mesh that is removed, the more significant the risks are. Similar to any operation in your pelvic area, there are general risks of significant bleeding, infection, delayed wound healing, deep vein thrombosis (also known as DVT which refers to clots in your legs or your lungs) and the anaesthetic risks. You will have wounds inside your vagina and possibly on your lower abdomen. If you had a trans-obturator procedure (TVT-O / TOT) with mesh placed through the obturator membrane (see diagram), you may have incisions in your groins if the aim is to remove the mesh completely.

The risks specific to the removal of the mesh are:

- **Damage to bladder, ureters, urethra or bowel**
  The mesh will either be already exposed into your bladder, ureters, urethra or bowel or it will be very close to these structures. If it is already exposed, that means there is a hole that will need to be fixed after the mesh is removed. If it is not exposed, there is a risk of damage that can happen during the removal of the mesh, and that damage will need to be repaired during the surgery. If the damage does not heal well, there is a risk of a permanent hole (fistula) that may need further surgery in the future. If there is a risk of damage to your urethra either by the mesh or during its removal, your surgeon may offer you a procedure to provide extra support to the urethra to help with the healing process.

- **Worsening pain**
  Any surgical procedure has a risk of causing pain. If the pain is related to scarring or irritation of nerves, it may become a long-term problem. Removing mesh would always involve operating in an area that is already scarred, and so there is a risk of worsening scarring and nerve injury and therefore worsening or new onset pain. If you have wounds in your groins to remove the mesh, there is a risk of bruising and injury to the nerves in your thighs, which may cause pain.

- **Worsening or recurrence of prolapse / incontinence**
  The reason you had the mesh inserted would have been to treat your incontinence or prolapse. The more mesh is removed, the higher the risk that the support is lost and your initial symptoms come back and may be worse than before. Sometimes, scar tissue will continue to give enough support even when the mesh is removed, and you may have no problems. But there is a risk that you may need further surgery in the future, if you are bothered by incontinence or prolapse again.

- **Failure to remove the mesh completely**
  Depending on the position of the mesh, it may not be possible to remove it completely without significant risk of injury. Especially with the trans-obturator meshes (TVT-O / TOT), it may not be possible to remove them completely without significant trauma to the thighs. This may then cause pain and possibly problems walking. However, for symptom control, it may be sufficient to remove the majority of the mesh, which is accessible through an incision in the vagina. It will be very important to discuss the risks and benefits in your individual case with your surgeon in detail to decide whether the risks of incisions in your groins would be of any benefit to you.

If you are considering having your mesh removed, it is very important to carefully consider the risks and expected benefits before you undergo this procedure.

If any surgery is considered or needed, your personal case will first be discussed within a multi-disciplinary team (MDT). Several specialists will have input into the advice that is given to you before you decide how to go forward. You may also want to speak to your GP and you may like to hear the opinions of more than one specialist.

Where can I find more information?

For further information you may like to access the following websites:

International Urogynaecology Association (IUGA): https://www.iuga.org/

British Society for Urogynaecology (BSUG): https://bsug.org.uk/

British Association of Urological Surgeons (BAUS): https://www.baus.org.uk/

If you do not have access to the internet, please discuss with your GP and ask them to provide a hard copy of relevant information you may like to see.

Disclaimer:

The risk levels quoted are according to available evidence as reported in the medical literature at the time of writing.

Authors:

Dr J. Wilkens, Miss V. Granitsiotis and Scottish Trans-vaginal Mesh Oversight (TVMO) Group.

Date:

May 2020

Medical Photography Service, NHS Lothian, June 2020. MD.
